# Intracellular Reprogramming of Expression, Glycosylation, and Function of a Plant-Derived Antiviral Therapeutic Monoclonal Antibody

**DOI:** 10.1371/journal.pone.0068772

**Published:** 2013-08-15

**Authors:** Jeong-Hwan Lee, Da-Young Park, Kyung-Jin Lee, Young-Kwan Kim, Yang-Kang So, Jae-Sung Ryu, Seung-Han Oh, Yeon-Soo Han, Kinarm Ko, Young-Kug Choo, Sung-Joo Park, Robert Brodzik, Kyoung-Ki Lee, Doo-Byoung Oh, Kyung-A Hwang, Hilary Koprowski, Yong Seong Lee, Kisung Ko

**Affiliations:** 1 Department of Medicine, Medical Research Institute, College of Medicine, Chung-Ang University, Seoul, Korea; 2 Korean Research Institute of Bioscience and Biotechnology, Daejeon, Korea; 3 Department of Biological Science, Biotechnology Institute, College of Natural Science, Wonkwang University, Iksan, Korea; 4 Department of Agricultural Biology, College of Agriculture and Life Science, Chonnam National University, Gwangju, Korea; 5 Department of Neuroscience, School of Medicine, Konkuk University, Seoul, Korea; 6 Department of Herbology, School of Oriental Medicine, Wonkwang University, Iksan, Korea; 7 Biotechnology Foundation Laboratories, Thomas Jefferson University, Philadelphia, Pennsylvania, United States of America; 8 National Veterinary Research and Quarantine Service, Anyang, Korea; 9 Department of Agrofood Resources, National Academy of Agricultural Science, RDA, Suwon, Korea; 10 Department of Urology, College of Medicine, Kangnam Sacred Heart Hospital, Hallym University, Seoul, Korea; The Scripps Research Institute and Sorrento Therapeutics, Inc., United States of America

## Abstract

Plant genetic engineering, which has led to the production of plant-derived monoclonal antibodies (mAb^P^s), provides a safe and economically effective alternative to conventional antibody expression methods. In this study, the expression levels and biological properties of the anti-rabies virus mAb^P^ SO57 with or without an endoplasmic reticulum (ER)-retention peptide signal (Lys-Asp-Glu-Leu; KDEL) in transgenic tobacco plants (*Nicotiana tabacum*) were analyzed. The expression levels of mAb^P^ SO57 with KDEL (mAb^P^K) were significantly higher than those of mAb^P^ SO57 without KDEL (mAb^P^) regardless of the transcription level. The Fc domains of both purified mAb^P^ and mAb^P^K and hybridoma-derived mAb (mAb^H^) had similar levels of binding activity to the FcγRI receptor (CD64). The mAb^P^K had glycan profiles of both oligomannose (OM) type (91.7%) and Golgi type (8.3%), whereas the mAb^P^ had mainly Golgi type glycans (96.8%) similar to those seen with mAb^H^. Confocal analysis showed that the mAb^P^K was co-localized to ER-tracker signal and cellular areas surrounding the nucleus indicating accumulation of the mAb^P^ with KDEL in the ER. Both mAb^P^ and mAb^P^K disappeared with similar trends to mAb^H^ in BALB/c mice. In addition, mAb^P^K was as effective as mAb^H^ at neutralizing the activity of the rabies virus CVS-11. These results suggest that the ER localization of the recombinant mAb^P^ by KDEL reprograms OM glycosylation and enhances the production of the functional antivirus therapeutic antibody in the plant.

## Introduction

Rabies virus causes a neuroinvasive disease that is typically fatal in humans. After the penetration of the virus and the subsequent onset of the associated clinical symptoms, there is no effective treatment. Recombinant rabies virus vaccines provide an effective method for the prevention of virus infections [1], [2]. However, after a rabies exposure, the currently recommended intervention strategy is to neutralize and clear the virus with antibodies or immunoglobulins (IgGs) through post-exposure prophylaxis (PEP) before the virus enters the nervous system. The use of human or equine rabies immune globulin has saved the lives of countless patients who would have died if treated with vaccine alone. Unfortunately, the worldwide shortage of the IgGs has hampered global efforts to provide PEP against rabies [3].

Typically, recombinant pharmaceutical proteins such as antibodies and therapeutic proteins are produced in animal systems. Alternatively, keep plant systems can be used for the large-scale production of these proteins. Plant systems offer several advantages including low upstream cost inputs, an absence of human or animal pathogen contaminants, and the ability to employ post-translational modifications such as glycosylation [4]–[8]. Many therapeutic and diagnostic mAbs have been expressed successfully in plants, including full-length IgGs, Fab fragments, single variable domains, antibody-fusion proteins, and single-chain antibodies [5], [9], [10].

The biosynthesis of *N*-linked glycans in plants differs from that of mammalian cells [11]. Although plants synthesize complex *N*-linked glycans containing a core Man_3_GlcNAc_2_ that bears 2 terminal *N*-acetylglucosamine (GlcNAc) residues, which are similar to those found in mammals, a β(1,2)-xylose (Xyl), Lewis^a^ epitopes, and an α(1,3)-fucose (Fuc) exist on the Man_3_GlcNAc_2_ core in plants. These plant-specific epitopes are absent on mammalian glycans and are therefore recognized by allergen-reactive mammalian IgEs [12], [13].

Glycoproteins are *N*-glycosylated in the ER and the Golgi complex and then secreted into subcellular compartments such as vacuoles and the extracellular space. Glycosylation processing in the ER is conserved amongst almost all species and restricted to OM (Man_5–9_GlcNAc_2_)-type glycans, whereas the Golgi-generated glycans are highly diverse [14]. In plants, the addition of KDEL at the C-terminal end of a protein is sufficient for the protein to be retained in the ER [15], [16]. mAb^P^s with KDEL fused to their heavy chain (HC) and light chain (LC) therefore contain exclusively non-immunogenic, OM type glycans with stable ER accumulation [17]. Gradinaru *et al.* [18] found that their protein of interest accumulated in the ER when it contained the KDEL in mammalian cells, and this ER retention of proteins in plants usually improved the production levels [19], [20]. However, an *in vivo* study in mice demonstrated that the anti-rabies mAb^P^ with OM type glycans was cleared from serum more rapidly than mAb^H^ [5]. The rapid clearance might be due to a number of possibilities, including immunogenicity resulting from KDEL itself acting as an epitope, a glycan residue-derived conformational alteration of the Fc domain [21], the OM structure being easily accessible to Man binding lectin [22], and a lack of terminal sialylation, which contributes to protein instability [23]. It has not been clearly understood whether the shorter half-life is due to the OM or lack of sialylation on the glycoproteins [5]. Unlike mAbs for cancer therapy, an anti-rabies mAb for PEP with rapid clearance is beneficial because interference between the mAbs and the vaccine can then be avoided [5].

In the present study, we expressed and characterized a human anti-rabies mAb derived from plants with or without the C-terminal KDEL tag for ER retrieval and demonstrated its effectiveness *in vitro* and *in vivo*. Both mAb^P^K and mAb^P^ were compared with the mAb^H^ for the rabies virus in their expression level, ER localization, *N*-glycan processing, neutralization activity, and protein stability. The KDEL-tagged mAb became predominantly localized in the ER, thus enhancing the mAb assembly in the plant cells. Therefore, the KDEL tagging to the mAb helped to enhance the final mAb yield in plants.

## Results

### Expression of mAb SO57 in Tobacco Transgenic Plants

**Figure 1:**
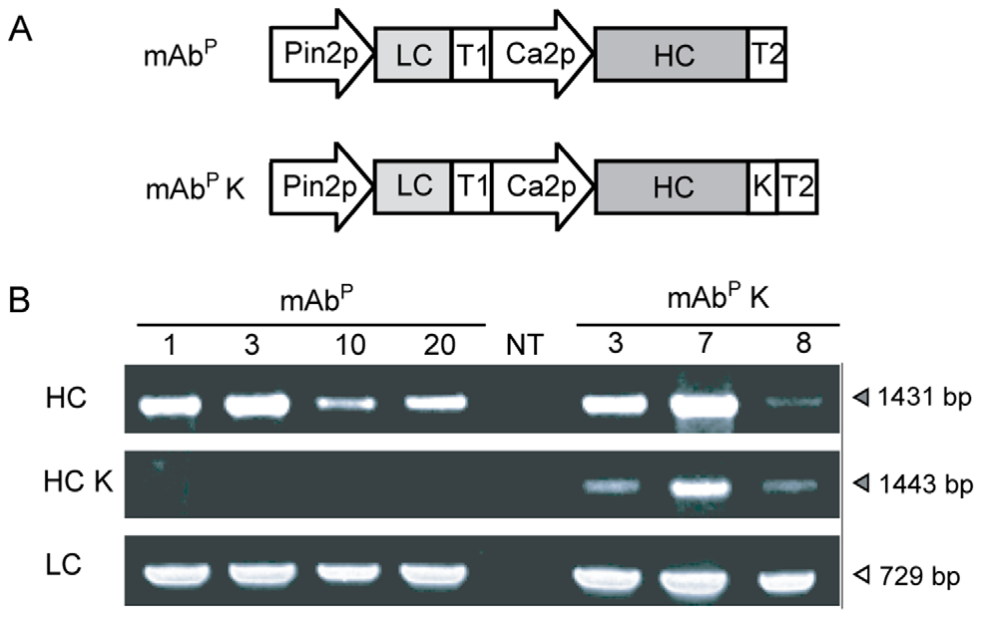
Expression of mAb SO57 in transgenic plants. (A) A schematic diagram of the mAb SO57 HC and LC DNA constructs [7]. (B) PCR analysis of LC (729 bp), HC (1431 bp) and HC fused to KDEL (HCK, 1443 bp) in the genomic DNA. NT, non-transgenic plant; mAb^P^, non-KDEL-tagged mAb^P^; mAb^P^ K, KDEL- tagged mAb^P^.

Transgenic tobacco lines were obtained by *Agrobacterium*-mediated transformation with plant expression vectors carrying HC, HCK, and LC of the human mAb SO57. Both HC and HCK cDNA were placed downstream of the alfalfa mosaic virus untranslated leader sequence (AMV) under the control of the enhanced cauliflower mosaic virus 35S promoter (*Ca2p*), while LC cDNA was under control of the potato proteinase inhibitor II gene (*Pin2*) promoter (Figure 1A). PCR amplification was conducted to confirm the presence of HC, HCK, and LC genes in genomic DNA isolated from the randomly selected transgenic plants (Figure 1B). The amplified HC (1431 bp), and LC (729 bp) fragments were detected in all samples, whereas the HCK (1443 bp) genes were detected only in the transgenic plants with HCK (Figure 1B). None of the transgenes were detected in the non-transgenic plants.

### Evaluation of mAb SO57 HC and LC Expression Levels by Real-Time Quantitative Reverse Transcription-PCR (RT-qPCR) and Immunoblotting

**Figure 2:**
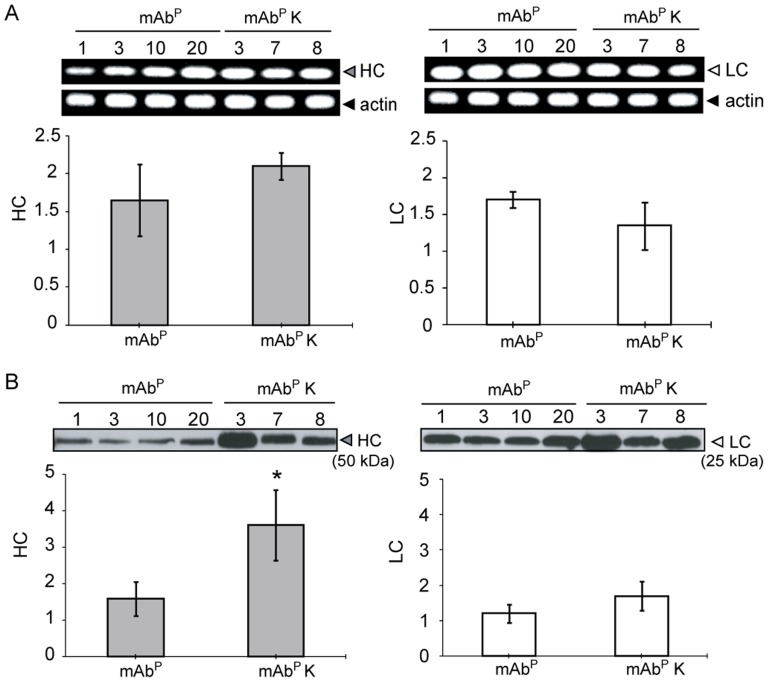
The RT-qPCR and immunoblot analysis of mAb SO57 HC and LC in transgenic tobacco plants. (A) Relative expression levels (Y axis) of the HC and LC gene as determined by RT-qPCR. Amplicons generated with the RT-qPCR were confirmed by 1% agarose gel electrophoresis (upper panel). The results of RT-qPCR are expressed as the average of three independent experiments after normalization with *N. tabacum* actin bands (lower panel). Error bars represents mean relative expression values (mean ± SD) of HC and LC to actin from the mAb^P^ and mAb^P^K samples. Transcript levels of HC and LC were not significantly different between the mAb^P^ and mAb^P^K (*p*>0.05). mAb^P^, non-KDEL-tagged mAb^P^; mAb^P^K, KDEL-tagged mAb^P^. (B) Western blot analysis of proteins extracted from leaves of randomly selected 4 transgenic plants without KDEL and 3 transgenic plants with KDEL, respectively. The bands for HC (50 kDa) and LC (25 kDa) were detected with HRP-conjugated goat anti-human Fcγ- or F(ab′)_2_-specific antibodies, respectively. *p<0.05 compared to mAb^P^ samples (Student's t-test analysis). Error bars represent the mean ± SD.

A RT-qPCR assay was performed to determine the transcription levels of HC and LC in randomly selected transgenic plants (Figure 2A). RT-qPCR products of the expected sizes for the mAb SO57 HC (67 bp; Left) and LC (66 bp; Right) were detected in the transgenic plants (Figure 2A). When normalized against actin mRNA, transcript levels of both HC and LC were not significantly different between mAb^P^ and mAb^P^K (*p*>0.05) (Figure 2A). The expression of the HC and LC proteins in the transgenic plants was confirmed by immunoblot (Figure 2B). Both the HC (50 kDa) and LC (25 kDa) bands were identified in the total leaf soluble protein extracts from the transgenic plants by the anti-human Fcγ- and anti-F(ab′)_2_-specific secondary IgGs, respectively. In the immunoblot with the anti-human Fcγ-specific IgG, HC was detected in both mAb^P^ and mAb^P^K samples. In the immunoblot with the anti-F(ab′)_2_-specific IgG, LC was detected in all transgenic samples. In addition, the expression levels of HC (Left) and LC (Right) were higher in the mAb^P^K sample than that in the mAb^P^ sample (Figure 2B).

### Localization of mAb^P^ and mAb^P^K in Plant Cells

**Figure 3:**
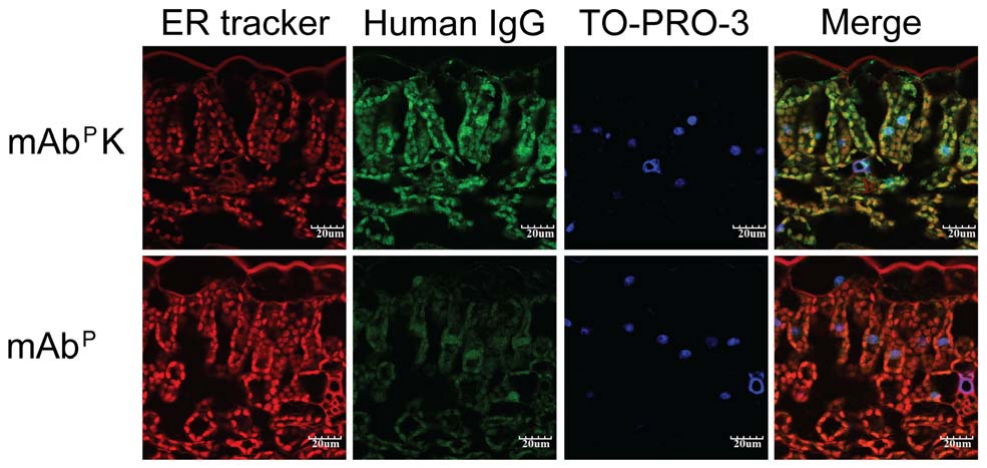
Confocal analysis of the subcellular localization of mAb SO57 and mAb SO57 K in plant leaves. Immunofluorescent confocal microscopic photomicrographs displayed localization of mAb SO57 in subcellular organelles of transgenic tobacco plant leaf cells. The red signal for ER-Tracker Blue-White DPX, a photo-stable probe selective for the ER in live cells, shows specific ER localization of mAb. The mAb SO57-immunoreactive green fluorescence was detected by FITC-conjugated anti-human IgG (green). The nuclei (blue) were labeled with TO-PRO-3. Each image was merged to analyze the subcellular localization of mAb SO57 in transgenic plants. The green fluorescent signal of mAb SO57 K closely overlapped with the blue fluorescent signal of ER-Tracker. Scale bars, 20 µm.

The ER, labeled red with ER tracker was observed in cells of both mAb^P^ and mAb^P^K transgenic plants (Figure 3, ER-tracker). mAb^H^ SO57-immunoreactive green fluorescence was seen in cells from mAb^P^ and mAb^P^K transgenic tobacco plants (Figure 3, and S1, Human IgG, respectively) whereas no green signal was found in cells from non-transgenic plants (NT) (Figure S1, Human IgG, lower panel). The nuclei, which were labeled blue with TO-PRO-3, were observed in cells from all of the plant samples (Figure 3, TO-PRO-3). In the mAb^P^K transgenic plant, the strong green fluorescent signal of antibody closely overlapped with the red fluorescent signal of ER-Tracker in round shape (Figure 3, Human IgG and Merge, upper panel) whereas in the mAb^P^ transgenic plant, the green fluorescent signal was roughly spread in cells without close overlapping to the red fluorescent signal (Figure 3, Human IgG and Merge, lower panel).

In the mAb^P^K transgenic plant, the antibody immunoreactivity disclosed a concentric green ring (Figure S1, Human IgG and Merge, upper panel, arrow heads) whereas in the mAb^P^ transgenic plant, a strong green ring shape was not observed (Figure S1, Human IgG and Merge, middle panel). In the mAb^P^K transgenic plants, the merge of green and blue showed, surrounding the outside of the blue-labeled nucleus, a relatively strong green ring, where the ER is distributed (Figure S1, Merge, upper panel). In contrast, in the mAb^P^ transgenic plants, the green did not surround the outside of the nucleus in the plant cells. In the non-transgenic plants, the nuclei labeled in blue were observed whereas the green was not detected (Figure S1).

### Purification of Plant-derived mAb SO57

**Figure 4:**
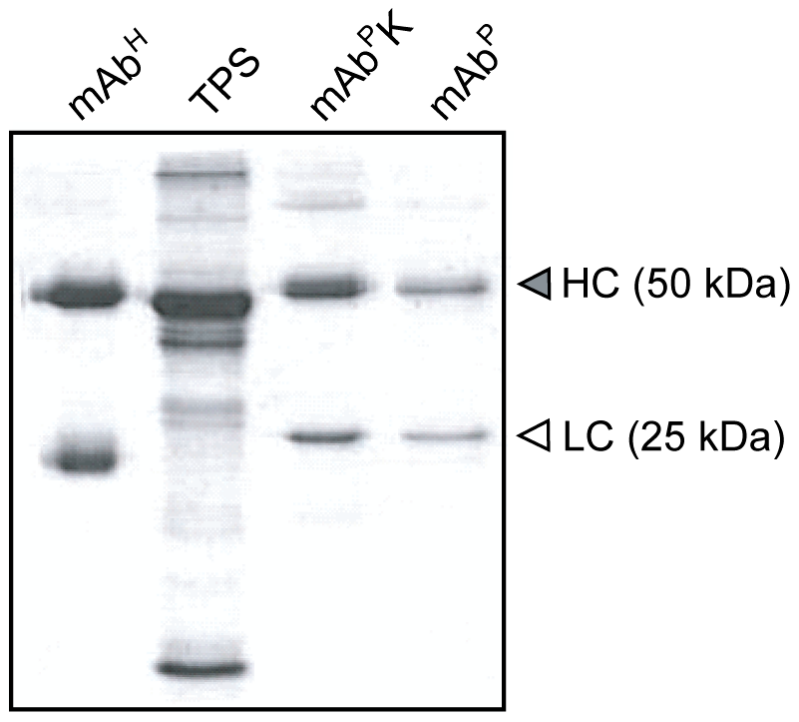
SDS/PAGE of the mAb SO57 purified from transgenic plants. Purified mAbs were loaded onto the gels and stained with Coomassie brilliant blue R250. TPS, total protein extract from plant leaves; mAb^H^, hybridoma-derived mAb SO57; mAb^P^, non-KDEL-tagged mAb^P^; mAb^P^K, KDEL-tagged mAb^P^; HC, heavy chains of mAb^P^; LC, light chain of mAb^P^.

mAbs were purified from leaves harvested from mAb^P^ and mAb^P^K transgenic tobacco plants. The protein A column purification yielded an average of 0.4 and 1.2 mg of mAb^P^ and mAb^P^K per kilogram of fresh leaves from high mAb expressing lines, respectively. SDS/PAGE analysis of the purified mAb^P^ revealed two major bands (50 and 25 kDa for the HC and LC, respectively) (Figure 4). The LC of mAb^P^ was slightly heavier than that of mAb^H^ (Figure 4). The discrepancy of mobility is due to the different buffer composition of purified samples between plant and human or the different signal peptide used to espress the recombinant LC gene in transgenic plants. In this study, different glycosylation is not the case since the LC does not have glycosylation sites according to amino acid sequences.

### Comparison of *N*-Glycan Structure and Neutralizing Activity of Purified mAb^P^ and mAb^P^K SO57

**Figure 5:**
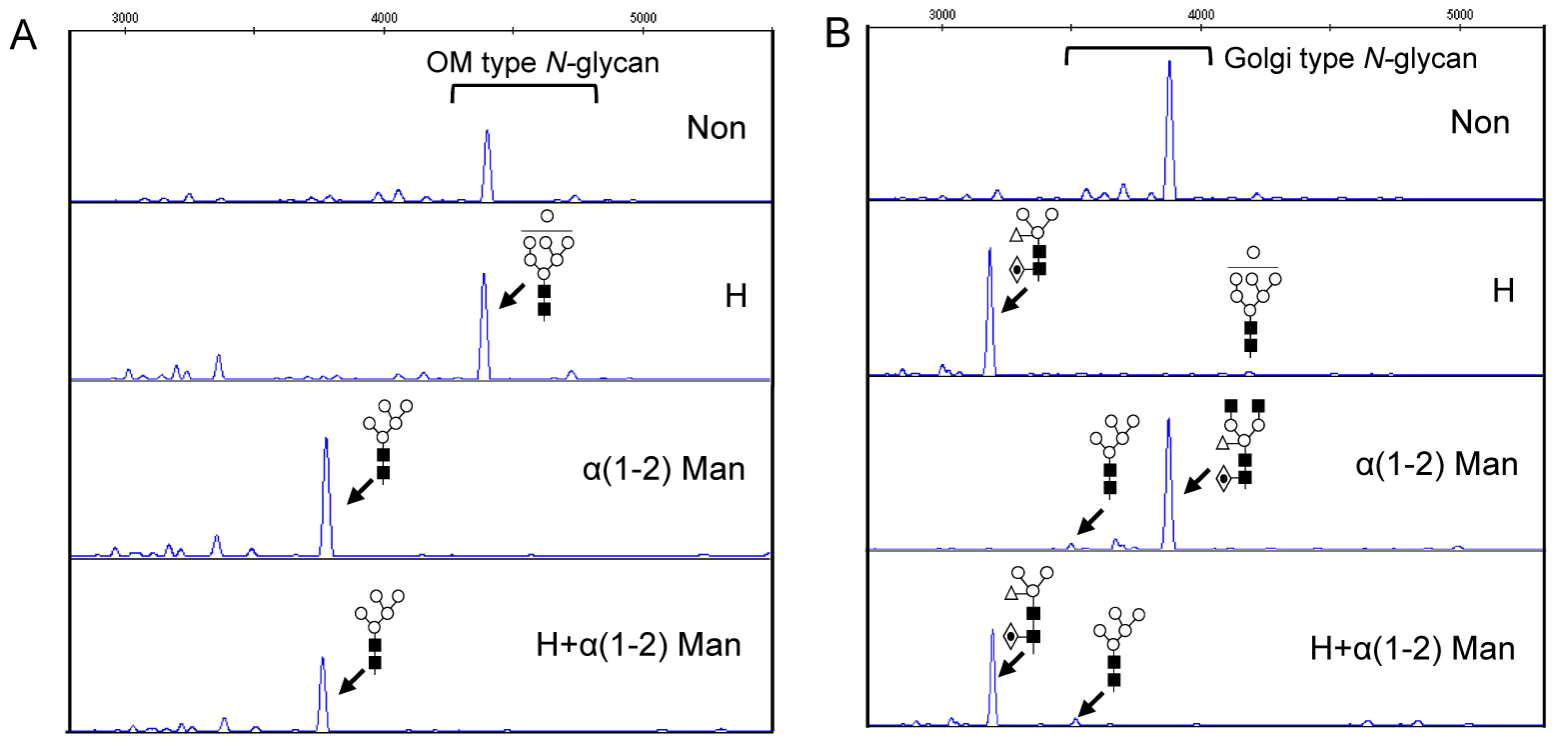
*N*-glycan profiles of anti-rabies mAb^P^K and mAb^P^. mAbs were expressed from transgenic tobacco plants as dual forms with mAb^P^K or mAb^P^. Glycan profiles of mAb^P^ (A) and mAb^P^K (B) were obtained by DNA sequencer-based method for the plant *N*-glycan analysis. Golgi-type and OM-type glycans were classified and confirmed by hexosamindase (H) and α(1,2)-mannosidase (α1,2-Man) digestions. The symbols of the glycan structures are as follow: GlcNAc, black square; Man, white circle; Xyl, white triangle; Fuc, diamond with a dot inside. Non, non-digested sample.

A DNA sequencer-based analysis was performed to compare the *N*-glycan profiles of mAb^P^, mAb^P^K, and mAb^H^ (Figure 5, S2, and S3). APTS (8-amino-1,3,6-pyrenetrisulfonic acid)-labeled glycan profiles are shown in Figure 5, and the peaks were identified by exoglycosidase digestion and by the comparison to glycan profiles previously assigned. Relative percents (%) for OM and Golgi types were calculated from the sum of the corresponding peak areas (Table 1). As expected, mAb^P^ had very low percentage of OM-type glycans. In contrast, mAb^P^K included a high portion of OM-type glycans (91.7%) together with Golgi-type glycans (8.3%). The mAb^H^ displayed a range of complex glycans, most of which (90%) contained a core α(1,6)-Fuc (Figure S2). In addition, an *in vitro* comparison of the neutralizing activity of mAb^P^ and mAb^P^K against cell culture-adapted rabies virus (CVS-11) indicated that both mAb^P^ and mAb^P^K were as active against the CVS-11 as was mAb^H^ (Table 1).

**Table 1:** Comparison of profiled glycan of mAb^P^, mAb^P^K, and mAb^H^, and their virus-neutralizing activity against rabies virus CVS-11.

Sample	% of total peak area		Neutralizing Activity (IU/ml)
	Glogi type	OM type	
mAb^H^	∼90	∼10	1.5
mAb^P^	96.8	3.2	1.5
mAb^P^K	8.3	91.7	1.5

OM type, oligomannose type.

### Clearance of Plant-derived mAb SO57

**Figure 6:**
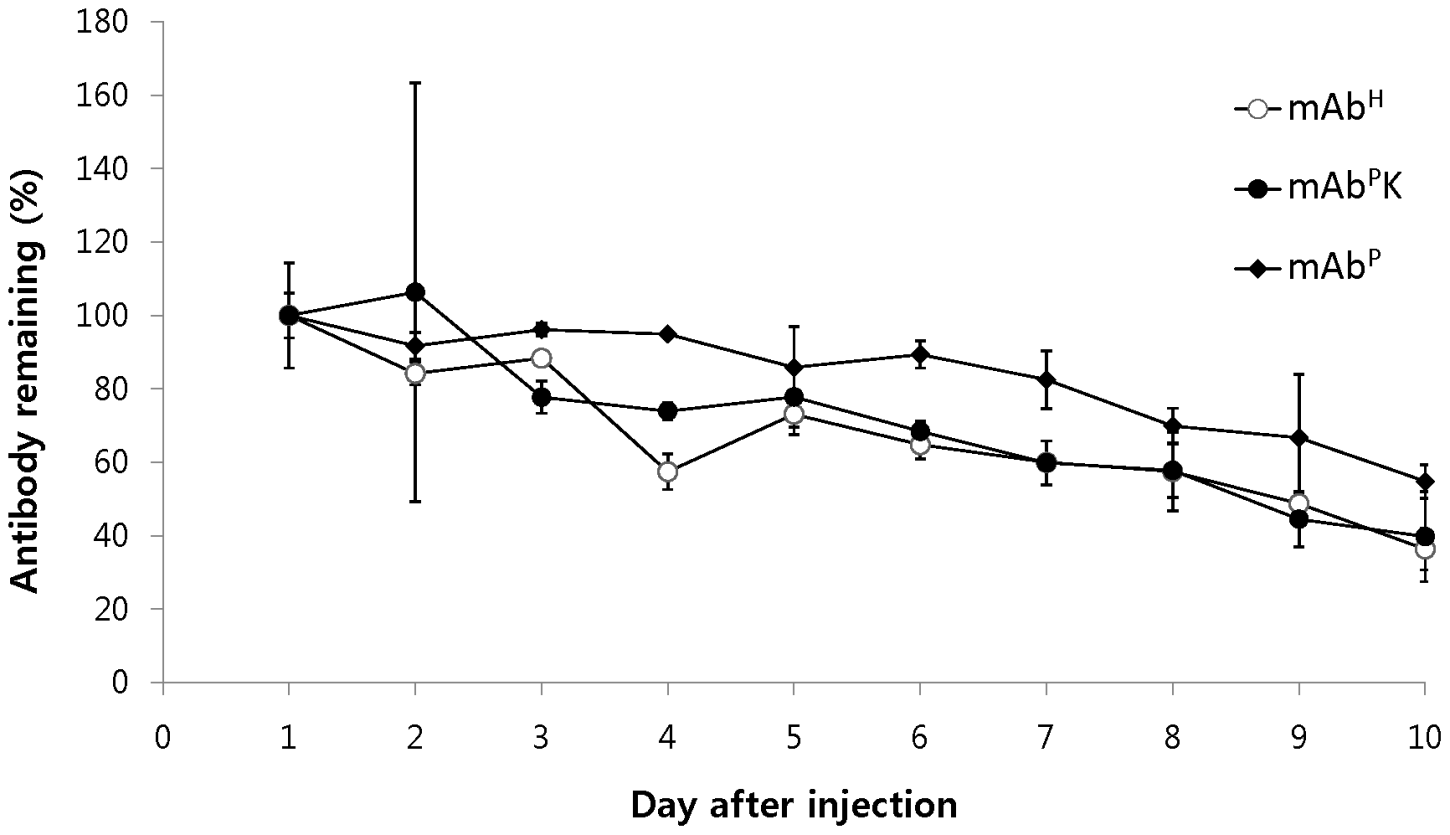
Stability profiles of mAb^H^, mAb^P^K and mAb^P^ in mice. The % value was calculated with the formula [100×(concentration of each day/concentration of the first day (24 hr) after injection)], followed up to day 10. The concentration of mAb in the serum from BALB/C mice injected i.p. with mAb^H^, mAb^P^K or mAb^P^ was determined by ELISA. Data are presented as means ± SD.

An *in vivo* comparative clearance test of mAbs was conducted in mice i.p. administered mAb^P^, mAb^P^K and mAb^H^. The blood samples were collected between days 1 and 10 after injection. Serum antibody concentrations were determined by ELISA. To exclude the possibility to miscalculate clearance rate due to difference in the initial antibody concentrations, the initial values were considered as 100% at day 1 after injection. The concentrations of antibodies in serum were expressed as relative percent (Figure 6). Between days 1 and 10, mAb concentrations slowly declined until day 10. The clearance trend of all three antibodies was similar between days 1 and 10. At day 10 after injection, the % value of mAb^H^ (36%) was statisticallty not different to mAb^P^K (39%)..

## Discussion

Our data demonstrate that plant cell reprogramming with the addition of an ER retention signal to mAb enhanced the expression of the rabies antibody, which had a virus neutralizing activity comparable to that of mAb^H^. In addition, the ER retention signal allowed control of the subcellular localization of the mAb and generated different glyco-structural patterns. The expression level of the mAb and its biological activities are essential elements for effective heterologous production of such a highly valuable therapeutic protein. In this study, two different plant expression vectors for the anti-rabies mAb with and without the KDEL (mAbK and mAb, respectively), which was fused to the HC, were designed in order to investigate the resulting expression levels and biological activities of the ER-retained and default secreted mAbs. The HC and LC were controlled under two different promoters, *Ca2p* and *Pin2p*, respectively, in order to avoid promoter-targeted transcriptional gene silencing [24]. The HCK had about 2 times greater protein accumulation than the HC without KDEL (HC). However, the relative transcription level of the HCK was not significantly different. These data indicate that the higher HC level in transgenic plants with mAb^P^K is due to ER accumulation of the mAb. The LC protein expression in transgenic plants with HCK was slightly higher compared to transgenic plants without KDEL, whereas the LC transcriptional level was relatively lower in HCK compared to HC transgenic plants. These results suggest that the LC protein was assembled together with the HCK, and consequently retained and accumulated in the ER. Taken together, in transgenic plants with HCK, protein expression levels were increased regardless of the similar transcription level, when compared to transgenic plants with HC. Thus, the KDEL can be applied to enhance the protein accumulation level [25].

The confocal analysis revealed that the mAb^P^K was localized around the outside of the nucleus where the ER is distributed [25], [26]. In contrast to the plants with HCK, the mAb^P^ did not surround the outside of the nucleus in plant cells. These observations are consistent with previous studies where proteins fused with KDEL were co-localized with ER surrounding the nucleus [18]. The glycan structures of the antibody can be altered by ER localization, and these alterations consequently impact on the antibody stability and function, such as the antibody-dependent cellular cytotoxicity (ADCC) [27].

**Figure 7:**
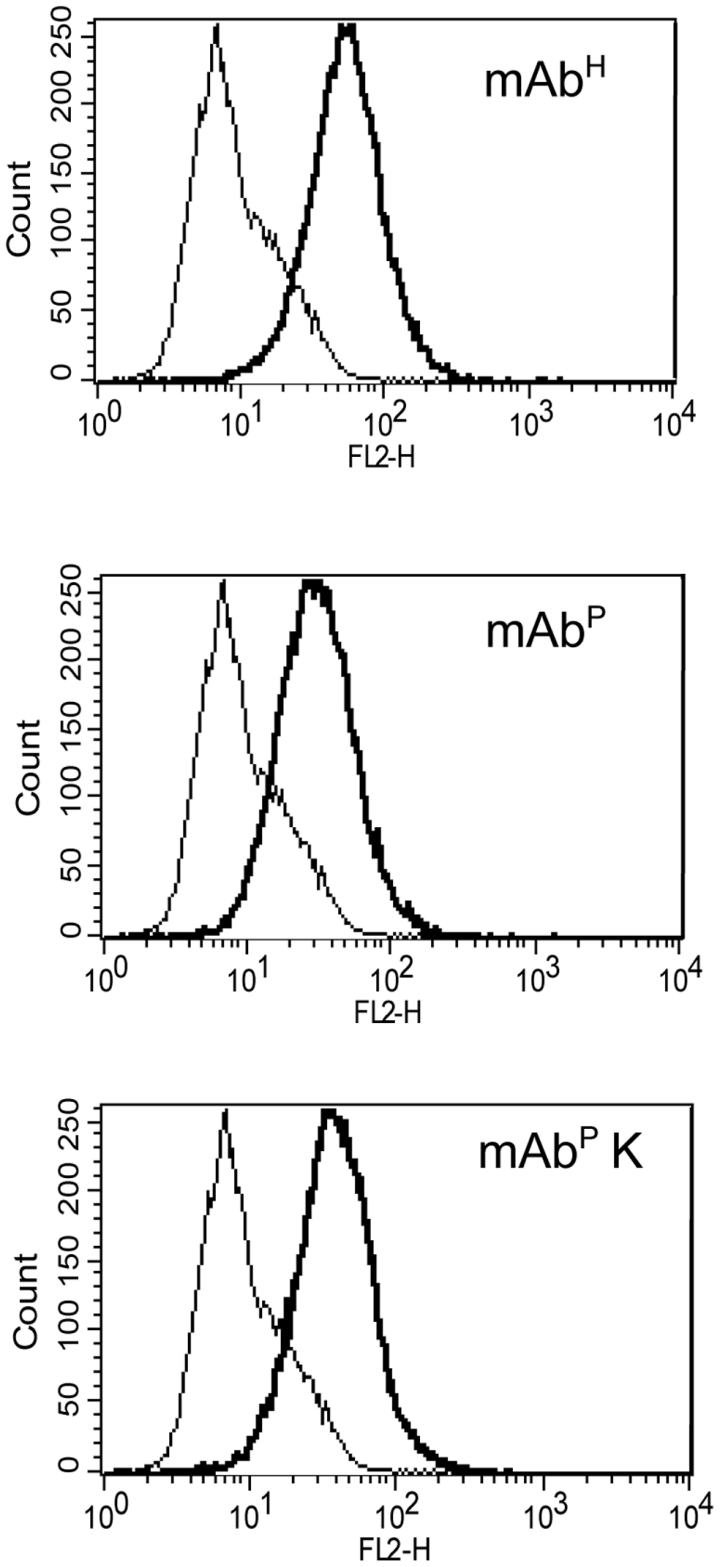
Flow cytometric analysis of binding activity of mAb^H^, mAb^P^, and mAb^P^ K SO57 to the FcγRI (CD64). U937 cells with IFN-γ to stimulate Fc receptor expression were incubated with mAb^H^, mAb^P^ or mAb^P^ K SO57, respectively. Binding activity of mAb^H^ (upper), mAb^P^ (center), and mAb^P^ K (bottom) to the activated cells expressing CD64 were analyzed by flow cytometry. Non-bold and bold lines indicate IFN-γ stimulated cells without any mAb, and with mAb^H^, mAb^P^ and mAb^P^K, respectively. The binding signals were detected with treatment of RPE-conjugated goat anti-human IgG.

In the glycosylation profiles of the mAbs analyzed using the DNA sequencer, mAb^P^K and mAb^P^ showed 91.7% and 3.2% of OM-type glycan structures, respectively, which indicates that the KDEL influences the glycan structure through the retention of the mAb in the ER and supports the present confocal analysis results. The mature glycan structures of plant proteins are characterized by the presence of β(1,2)-Xyl and/or α(1,3)-Fuc residues, which can cause allergenic and immunogenic responses [28], [29]. Thus, the KDEL has been added to the C-termini of proteins retained in the ER in order to yield OM-type glycans that avoid such glycoepitopes. Our previous studies demonstrated that OM-type glycans of mAb^P^K are associated with more rapid clearance *in vivo* compared with mAb^H^ [5]. It has been proposed that the increased clearance rate might be due to immunogenicity resulting from the KDEL itself acting as an epitope and/or due to the glycan residue-derived conformational alterations of the IgG Fc domain [21]. On the other hand, OM structures can be easily internalized into endocytic pathways in macrophages and dendritic cells upon which the surface carrying Man receptors bind the OM of mAb [30]–[32]. This internalization can be associated with faster clearance of circulating oligomannosylated antibodies [33]. In this study, however, both mAb^P^s, regardless of their OM or plant-specific complex type glycostructures had similar disappearance trends with mAb^H^. The mAb clearance trends observed in this study are similar to the previous report where plant-derived anti-hepatitis B virus mAbs with KDEL and without KDEL showed a similar clearance trend in mice from 1 to 10 day after injection [34]. These results suggest that the KDEL or OM glycan structures are not closely related to the clearance trend of mAb in mice sera. It is speculated that the non-sialylation of both mAb^P^s did not affect the faster clearance of mAbs in blood circulation regardless of OM or plant-specific complex glycan structures from 1 day to 10 day after injection. The interaction of the Fc portion of the antibody with the Fc receptor of the immune cells is essential to elicit ADCC, a mechanism of cell-mediated immunity whereby natural killer cells actively promote cell death in a target cell by triggering apoptosis. Previous studies have shown that anti-colorectal cancer mAbs with plant-specific glycostructures had similar *in vitro* interactions of Fc and the Fc Receptor I (CD64) to their parental mAb^M^ [7], [35] and *in vivo* anti-tumor activity [36]. In this study, the binding activities of mAb^P^, mAb^P^K and mAb^H^ to U937 cells (Human leukemic monocyte lymphoma cell line) [37] expressing the Fc Receptor I (CD64) were determined by flow cytometric assay (Figure 7). The FL2-H fluorescence peak (bold line) of mAb^P^K (bottom) were located similarly to the mAb^H^ (upper), whereas the peak (bold line) of mAb^P^ (center) was located slightly more to the left as compared to the peak (bold line) of mAb^H^ (upper). Binding activity to the Fc receptor is also essential for anti-rabies immunotherapy since the neutralization activity, which physically blocks the rabies virus particles by mAb SO57, is the only one required to acquire the anti-virus activity [38]. These results indicate that an OM-type of antibody can have slightly better interaction between Fc and Fc receptors compared to the plant-specific glycan type.

In this study, direct comparison of clearance time and biological activities of plant-derived anti-rabies virus monoclonal antibodies with high mannose and plant-specific glycan structure were performed, which has not been reported previously. Through these data, we now see that glycan structures might not significantly alter antibody stability. Certainly ER localization affects glycan structure and expression of recombinant mAbs in this plant expression system. The current results highlight the potential of OM glycomodifications of therapeutic anti-rabies virus mAbs produced in plants with KDEL-mediated subcellular ER localization. Even though mAb^P^ (plant-specific glycans) and mAb^P^K (OM-type glycans) had relatively similar virus neutralizing activity compared to mAb^M^, and thus similar potential for rabies PEP application, expression in the ER can overcome concerns about plant-specific glycoepitopes expressed by others [29] and provide the additional benefits of higher expression and relatively better effectiveness of Fc receptor-Fc interaction.

## Materials and Methods

### Ethics statement

BALB/c mice (female, 6–8 wk) were obtained from Daihan Biolink (Eumsung, Korea) and injected i.p. with plant-derived monoclonal antibody (mAb^P^) or hybridoma-derived monoclonal antibody (mAb^H^). After injection, blood samples were collected from the orbital sinus twice during the entire time period (10 days). The animal experiments were approved by the Institutional Animal Care and Use Committee (IACUC) of Wonkwang University, Iksan, Korea (Approval ID: WKU11-28). All efforts were made to minimize suffering of the animals.

### Plant Transformation Vector and Generation of Transgenic Plants

The mAb SO57 HC and LC genes were amplified by PCR with forward and reverse primers containing *Nco*I and *Xba*I, and *Pst*I and *BamH*I sites in the 5′ and 3′ ends, respectively. The HC and HCK cDNA were placed with AMV translational enhancer element under the control of the *Ca2p*, and the LC cDNA were under the control of the *Pin2p* in a pBI121-based plant expression vector (Figure 1A). The two different types of HC genes were cloned with and without fusion to the KDEL. Binary vectors for *Agrobacterium*-mediated plant transformation were obtained according to the protocol employed by a previous study [5]. The transgenic tobacco plants were selected on kanamycin (100 µg/ml), transplanted into soil, and maintained for subsequent generations.

### Polymerase Chain Reaction (PCR)

Genomic DNA was isolated from leaves by a DNeasy kit (Qiagen, Valencia, CA), according to the manufacturer's recommendations. PCR amplification was applied in order to confirm the presence of the genes encoding for mAb SO57 HC (1431 bp), LC (729 bp) and HCK (1443 bp) using the forward (F) and reverse (R) primers shown in Table S1. The PCR reaction was subjected to 30 cycles of 94°C for 20 sec, 53°C for 20 sec and 72°C for 90 sec. Non-transgenic plants were used as a negative control. Analysis of mRNA expression by RT-qPCR was performed as described in SI Materials and Methods.

### SDS-PAGE and Immunoblot Analysis

Leaf tissue (100 mg) was homogenized in Bradley buffer (50 mM Tris, 7.5 pH, 10 mM KCl, 20% glycerol, 0.4 M sucrose, 5 mM MgCl_2_, and 10 mM β-mercaptoethanol). The proteins in the homogenates were resolved by 12.5% SDS-PAGE and either stained by using Coomassie brilliant blue R250 or transferred to a nitrocellulose membrane (Millipore, Billerica, MA). Membranes were incubated in 3% skim milk (Fluka, Buchs, Switzerland) in PBS plus 0.5% (v/v) Tween 20 followed by goat anti-human Fcγ and F(ab′)_2_ fragment-specific antibodies conjugated to horseradish peroxidase (Jackson Immunolab, West Grove, PA) to detect HC and LC, respectively. Protein bands were visualized by exposing the membrane to X-ray film (Fuji, Tokyo, Japan) using a chemiluminescence substrate (Pierce, Rockford, IL). Non-transgenic plants and mAb^H^ SO57 (Bayrab, Bayer, Elkhart, IN) were used as negative and positive controls, respectively. Antibody levels were densitometrically analyzed using ImageQuant v2005 software (GE Healthcare, Freiburg, Germany).

### Confocal Fluorescence Analysis

Leaf samples were fixed for 5 days with 4% paraformaldehyde in 0.1 M phosphate buffer (pH 7.2). The fixed leaves were dehydrated with ethanol, cleared with xylene, and embedded in paraffin to make a paraffin block. The paraffin-embedded sample was sectioned into 10 µm slices and attached to gelatin-coated slides. The primary antibody (goat anti-human IgG; Animal Genetics, Daejeon, Korea) was applied to the coated slides, and the slides were then treated with green fluorescent Alexa-488 conjugated to a rabbit anti-goat IgG in order to detect the mAb SO57. The slide was then incubated with the nuclear stain TO-PRO-3 (Molecular Probes, Eugene, OR), which fluoresces blue at 633 nm.

### Purification of mAb^P^ SO57

Plant leaves (300 g) were homogenized on ice with extraction buffer (37.5 mM Tris-HCl, 50 mM NaCl, 15 mM EDTA, 75 mM sodium citrate, and 0.2% sodium thiosulfate) and centrifuged at 15,000×*g* for 30 min at 4°C. The supernatant was filtered through a Miracloth (Calbiochem, Darmstadt, Germany), and solid ammonium sulfate (AS) was added to produce a solution with 16% saturation. After 2 h of incubation at 4°C, the solution was centrifuged at 15,000×*g* for 30 min at 4°C, the precipitate was discarded, and AS was added to the supernatant to produce a solution with 40% saturation. After incubation at 4°C overnight, the solution was centrifuged as before, and the pellet was resuspended in an extraction buffer to one-fifth of the original volume. Soluble proteins were applied to a protein A column (GE Healthcare), and the mAb was eluted according to the manufacturer's recommendations. After overnight dialysis against 1× PBS, the mAb was concentrated by using an Amicon Ultra spin column with a 10 kDa cut-off (Millipore) and then stored at −80°C.

### *N*-glycan Analysis by DNA Sequencer

Purified mAbs were digested into glycopeptides with 0.1 µg of pepsin in 10 mM HCl buffer (pH 2.2) for 12 h at 37°C and then incubated for an additional 12 h after the addition of a second batch of 0.1 µg pepsin [39]. From the resulting glycopeptide mixtures, *N*-glycans were released using peptide *N*-glycosidase (PNGase) A (Roche, Mannheim, Germany). After the deglycosylation, the glycans were labeled by APTS and purified, as previously described [39]. The resulting APTS-labeled glycans were dissolved in 5 µl ultrapure water, and 1∶20 diluents were dispensed into a 96-well plate. The plate was loaded onto an ABI 3130 sequencer (Applied Biosystems, Foster City, CA) equipped with a standard 36 cm capillary array filled with POP-7 polyacrylamide linear polymer. The running parameters for the sequencer were the same as the previously described protocol [39], [40]. The resultant electropherograms were then analyzed using GeneMapper software (Applied Biosystems). *N*-glycan analysis by permethylation and mass spectrometry was performed as described in SI Materials and Methods.

### *In Vitro* Rabies Virus Neutralization Assay

The fluorescent antibody virus neutralization (FAVN) test was carried out as previously described [41]. Each mAb and standard reference serum containing a titer of 0.5 international units (IU)/ml was tested in 4 replicates in a 96-well tissue culture plate. Three-fold serial dilutions of mAb^P^, mAb^P^K, and mAb^H^ were incubated with the rabies virus cell cultured adapted standard strain (CVS-11) [41] for 60 min at 37°C. After incubation, a cell suspension containing 4×10^5^ BHK-21(baby hamster kidney cells) [42] cells/ml were added, and the mixture was allowed to incubate for 48 h at 37°C under 5% CO_2_. The plate was washed, fixed, and stained with the FITC-labeled anti-rabies mAb (Jeno Biotech, Chuncheon, Korea) and examined under a fluorescent microscope. Every well that showed specific fluorescence was considered to be positive. The median titer (D_50_) of the 4 replicate wells was calculated by the Spearman-Kärber formula. The titer of each mAb was expressed as IU/ml after comparison with that of standard serum.

### *In Vivo* Clearance of mAbs

BALB/c mice (female, 6–8 wk, n = 10 for each mAb, Daihan, Eumsung, Korea) were injected i.p. with 7 µg of mAb^P^, mAb^P^K, or mAb^H^ in 100 µl of 1× PBS buffer. After injection, blood samples were collected from the orbital sinus every day for 10 days; each mouse was bled twice during the entire time period. Serum levels of mAb^P^, mAb^P^K and mAb^H^ were detected by sandwich ELISA. Plates were coated with 2 µg/ml of rabbit anti-human IgG-Fc antibody (Bethyl Labs, Montgomery, TX) overnight at 4°C. The plates were then incubated with mouse serum at a dilution of 1∶100 (v/v) for 4 h and then with HRP-conjugated goat anti-human IgG Fcγ fragment-specific antibody (Jackson Immunolab) at a dilution of 1∶3,000 (v/v). The plates were treated with 3,3′,5,5′-tetramethylbenzidine (TMB) substrates in order to detect the signals for approximately 20 min, after which the signal was stopped with a TMB stop solution (KPL, Gaithersburg, MD). The antibody titers in 3 wells per tested serum were estimated by determining the optical densities at 450 nm using a Tecan ELISA reader.

### Flow Cytometric Analysis of mAb SO57 Binding to the IgG receptor, the FcγRI Receptor (CD64)

U937 human lymphoma cells were stimulated to express the CD64 with 300 units/ml of interferon (IFN)-γ (Boehringer Ingelheim, Biberach, Germany) overnight at 37°C [43]. The stimulated cells were then incubated for 1 h at 4°C with 10 µg/ml of purified mAb^H^ SO57, mAb^P^ SO57 or mAb^P^ K SO57 in PBS containing 1% BSA and 0.02% sodium azide (immunofluorescence buffer, IFB). R-phycoerythrin (RPE)-conjugated goat anti-human IgG (Southern Biotech, Birmingham, AL) was used to stain the human mAbs bound to CD64. Fluorescein isothiocyanate (FITC)-conjugated anti-human CD64 (eBioscience, San Diego, CA) was applied to confirm the surface expression of CD64 on IFN-γ stimulated U937 cells. Cells were washed twice with IFB and analyzed with a FACSCalibur flow cytometer (BD Biosciences, San Jose, CA),

### RT-qPCR

The mRNA expression of the mAb HC and LC was quantified by using RT-qPCR. Total RNA was extracted from leaves with TRIzol reagent according to the manufacturer's protocol. To remove genomic DNA, 600 ng of total RNA was treated using a TURBO DNA-free™ (Ambion, Austin, TX) kit in a reaction volume of 20 µl. Each RNA sample was used as a template for RT reactions performed with AccuPower RT/PCR PreMix (Bioneer, Daejeon, Korea). A total volume (20 µl) contained 120 ng of template DNA-free RNA and oligo d(T)16 primer and random hexamers. Primers for RT-qPCR were designed with the aid of Primer Express software (Applied Biosystems) using default parameters. The cDNA samples (2 µl) were used for the RT-qPCR reaction, and the quantification was conducted on the the StepOne™ Real-Time PCR System (Applied Biosystems) using Taqman 2× Universal PCR Master Mix, and primers and probe sets specific for the mAb SO57 HC and LC. The reporter FAM™ and nonfluorescent quencher BHQ™ dyes formed 5′ and 3′ modifications, respectively. A StepOne™ Real-Time PCR System was set for 1 cycle at 50°C for 2 min, 1 cycle at 95°C for 5 min, and 40 cycles of denaturation at 95°C for 15 sec and annealing/extension at 60°C for 1 min. A *Nicotiana tabacum* actin gene [44] was used as an endogenous control in the RT-qPCR reactions. The relative mAb SO57 HC and LC mRNA levels in each sample have been expressed as a ratio with the relative mRNA levels of actin for each sample. Primers used for RT-qPCR are listed in Table S2.

### N-glycan analysis by permethylation and mass spectrometry

Prior to mass spectrometric analysis, glycans were permethylated for enhancing sensitivity by solid phase permethylation using spin-column method [45]. First, dried glycans were dissolved in a mixture of 90 µl DMSO, 2.7 µl distilled water, 35 µl iodomethane. The resulting mixtures were passed eight times over a spin-column packed with sodium hydroxide mesh beads using the centrifugation at 400×g. After the washing step with acetonitrile, eluates were collected by adding 400 µl chloroform. One ml of 500 mM NaCl solution was added, mixed well and then the upper layer was carefully removed after centrifugation. This liquid-liquid extraction step was repeated twice. The chloroform layer containing permethylated glycans was dried and resuspended in 4 µl of 50% aqueous methanol solution for mass analysis. MALDI-TOF mass spectrometry was performed in the reflector positive ion mode using a Microflex TOF (Bruker Daltonik GmbH, Bremen, Germany). For preparation of matrix solution, DHB (2,5-dihydroxybenzoic acid) was dissolved at a 10 mg/ml concentration in 1 mM sodium acetate aqueous solution. Equal volumes of permethylated glycans in 50% methanol solution and the prepared matrix solutions were mixed and then applied onto a MALDI MSP 96 polished steel Chip (Bruker Daltonik). After drying, mass spectra were acquired with the method recommended by the manufacture.

### Statistical analysis

All experiments were repeated at least three times. Asterisks (*) in the figures indicate differences deemed significant (P<0.05) by a two-tailed Student's t test. Data were shown as mean ± SD.

## Supporting Information

Table S1 **Primers used for amplification of mAb SO57 HC, LC and HCK.**
(DOCX)

Table S2 **Primers and probes used in the RT-qPCR.**
(DOCX)

Figure S1 **Confocal analysis of the subcellular localization of mAb SO57 and mAb SO57 K in plant leaves.** Immunofluorescent confocal microscopic photomicrographs displayed localization of mAb SO57 in subcellular organelles of transgenic tobacco plant leaf cells. The mAb SO57-immunoreactive green fluorescence was detected by FITC-conjugated anti-human IgG (green). The nuclei (blue) were labeled with TO-PRO-3. Each image was merged to analyze the subcellular localization of mAb SO57 in transgenic plants. mAb^P^K, transgenic plant expressing mAb^P^K; mAb^P^, transgenic plant expressing mAb^P^; NT, non-transgenic plant; DIC, differential interference contrast image; Merge (DIC), Merge image merged with DIC. Arrow heads indicate a concentric green ring surrounding the nucleus. The bar represents 20 µm.
(TIF)

Figure S2 **N-glycan profiles of human-derived anti-rabies monoclonal antibodies (mAb^H^) obtained by DNA sequencer.** The symbols of the glycan structures are as follows: GlcNAc, black square; mannose, white circle; fucose, diamond; galactose diamond with a dot inside. Non, no glycosidase treatment; S, pre-treatment with α(2, 6, 8) sialidase; S+G, S pre-treatment with β(1–4) galactosidase; S+G+F, S+G pre-treatment with α(1–2, 3, 4, 6) fucosidase; S+G+F+H, S+G+F pre-treatment with of β-N-acetylhexosaminidase.
(TIF)

Figure S3 **N-glycan analysis by mass spectrometry.** Glycan profiles were cross-checked by mass spectrometric analysis, which provided the possible glycan structures. After permethylation for enhancing the sensitivity, the mass of glycans prepared from mAb^P^ K (A) and mAb^P^ (B) were analyzed. The symbols of the glycan structures are as follows: GlcNAc, black square; mannose, white circle; xylose, white triangle; fucose, diamond with a dot inside.
(TIF)
