# Supplementary material for: Intracellular Reprogramming of Expression, Glycosylation, and Function of a Plant-Derived Antiviral Therapeutic Monoclonal Antibody
Source: PLoS One. 2013 Aug 15;8(8):e68772. doi: 10.1371/journal.pone.0068772 (PMC3744537; doi:10.1371/journal.pone.0068772)
Supplement: Table S1 — Primers used for amplification of mAb SO57 HC, LC and HCK. (DOCX) [file pone.0068772.s001.docx]

**Table S1. Primers used for amplification of mAb SO57 HC, LC and HCK.**

| Primers | | Sequences |
| --- | --- | --- |
| HC | Forward | 5′-CGCCATGGACTGGACCTGGAGGTTC-3′ |
| HC | Reverse | 5′-GCTCTAGATTAGTGATGGTGATGGTGATGTT TACCCGGGGACAGGGAG-3′ |
| HCK | Reverse | 5´-GCTCTAGATTAGAGCTCATCTTTGTGATGGTGA TGGTGATGTTTACCCGGGGACAGGGAG-3´ |
| LC | Forward | 5′-cgggatccatgagtgtccccaccatggcc-3′ |
| LC | Reverse | 5′-cgctgcagctatgaacattctgtaggggc-3′ |
