# Supplementary material for: Intracellular Reprogramming of Expression, Glycosylation, and Function of a Plant-Derived Antiviral Therapeutic Monoclonal Antibody
Source: PLoS One. 2013 Aug 15;8(8):e68772. doi: 10.1371/journal.pone.0068772 (PMC3744537; doi:10.1371/journal.pone.0068772)
Supplement: Table S2 — Primers and probes used in the RT-qPCR. (DOCX) [file pone.0068772.s002.docx]

**Table S2. Primers and probes used in the RT-qPCR.**

| Target gene | Accession no. | Sequences of primers | PCR product  (bp) |
| --- | --- | --- | --- |
| Heavy chain | AY172957 | HC-F 5´-TCCAGGGCAGACTCACCATT-3´ |  |
|  |  | HC-R 5´-AGGCTGCTCAGCTCCATGTAG-3´ | 67 |
|  |  | Probe 5´-FAM-CCGCGGACGAATCCACGAGC-BHQ-3´ |  |
| Light chain | AY172960 | LC-F 5´-CCACCACACCCTCCAAACA-3´ |  |
|  |  | LC-R 5´-CAGGCGTCAGGCGCAGGGTA-3´ | 66 |
|  |  | Probe 5´-FAM-AGCAACAACAAGTACGCGGCCA GC-BHQ-3´ |  |
| Actin | X69885 | Actin-F 5´-GCTGAGCGTTTCCGTTGTC-3´ |  |
|  |  | Actin-R 5´-GATTCCGGCAGCTTCCATT-3´ | 69 |
|  |  | Probe 5´-FAM-TGAGGTCCTTTTCCAACCATCAA TGATTG-BHQ-3´ |  |

F, forward primer; R, reverse primer
