# Supplementary figures and images for: Intracellular Reprogramming of Expression, Glycosylation, and Function of a Plant-Derived Antiviral Therapeutic Monoclonal Antibody
Source: PLoS One. 2013 Aug 15;8(8):e68772. doi: 10.1371/journal.pone.0068772 (PMC3744537; doi:10.1371/journal.pone.0068772)

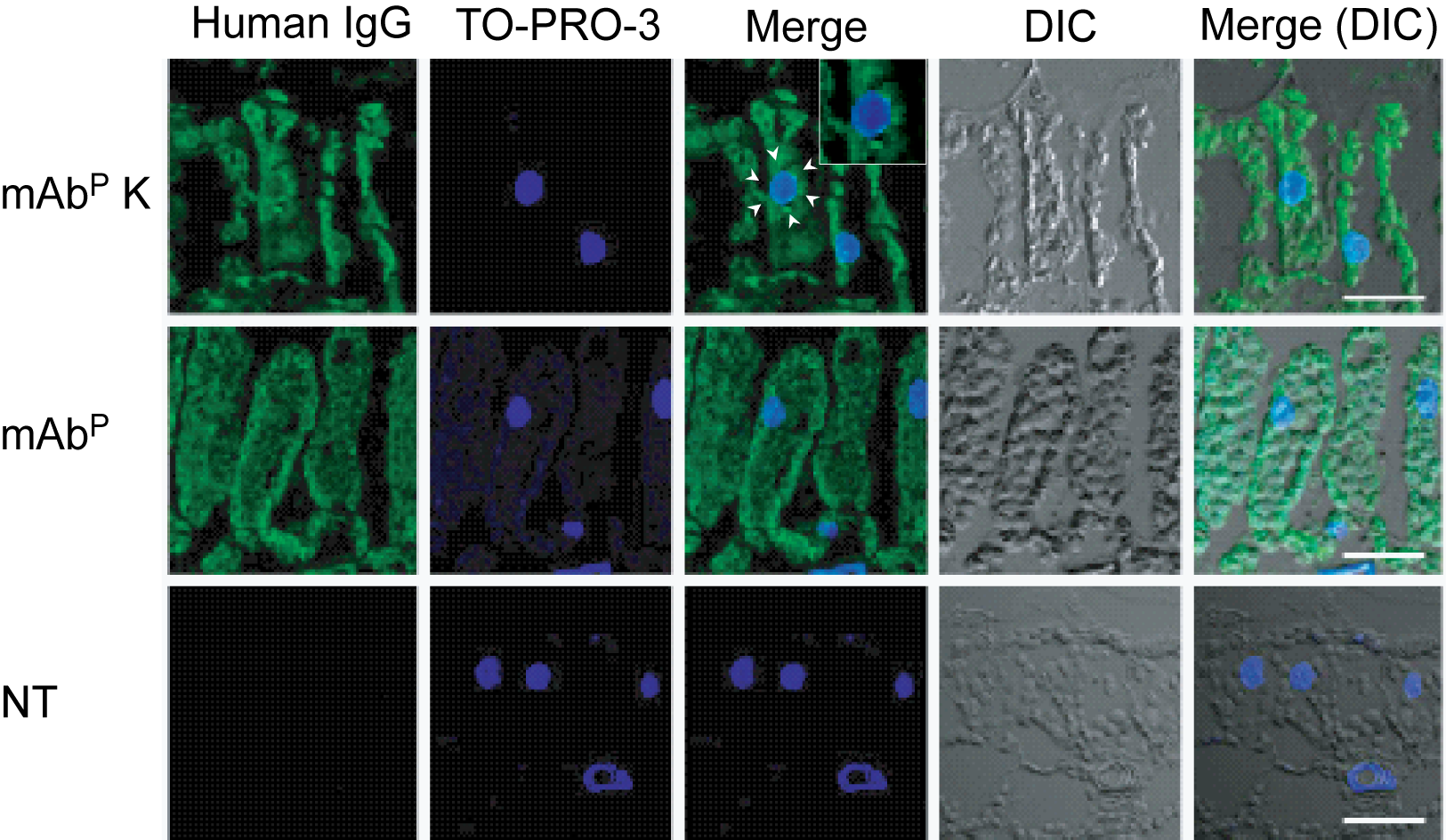

Supplement: Figure S1 — Confocal analysis of the subcellular localization of mAb SO57 and mAb SO57 K in plant leaves. Immunofluorescent confocal microscopic photomicrographs displayed localization of mAb SO57 in subcellular organelles of transgenic tobacco plant leaf cells. The mAb SO57-immunoreactive green fluorescence was detected by FITC-conjugated anti-human IgG (green). The nuclei (blue) were labeled with TO-PRO-3. Each image was merged to analyze the subcellular localization of mAb SO57 in transgenic plants. mAbPK, transgenic plant expressing mAbPK; mAbP, transgenic plant expressing mAbP; NT, non-transgenic plant; DIC, differential interference contrast image; Merge (DIC), Merge image merged with DIC. Arrow heads indicate a concentric green ring surrounding the nucleus. The bar represents 20 µm. (TIF) [file pone.0068772.s003.tif]

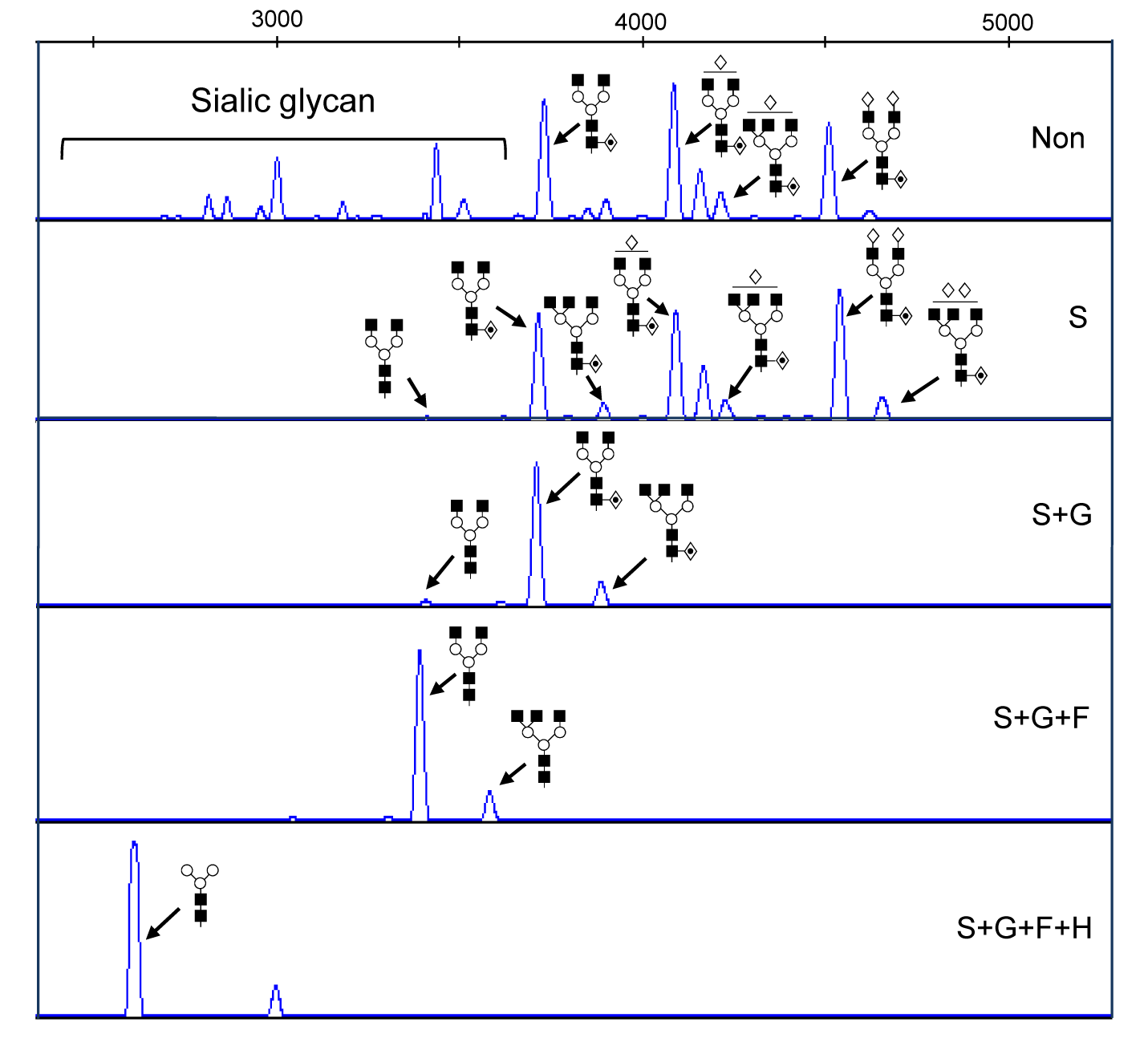

Supplement: Figure S2 — N-glycan profiles of human-derived anti-rabies monoclonal antibodies (mAbH) obtained by DNA sequencer. The symbols of the glycan structures are as follows: GlcNAc, black square; mannose, white circle; fucose, diamond; galactose diamond with a dot inside. Non, no glycosidase treatment; S, pre-treatment with α(2, 6, 8) sialidase; S+G, S pre-treatment with β(1–4) galactosidase; S+G+F, S+G pre-treatment with α(1–2, 3, 4, 6) fucosidase; S+G+F+H, S+G+F pre-treatment with of β-N-acetylhexosaminidase. (TIF) [file pone.0068772.s004.tif]

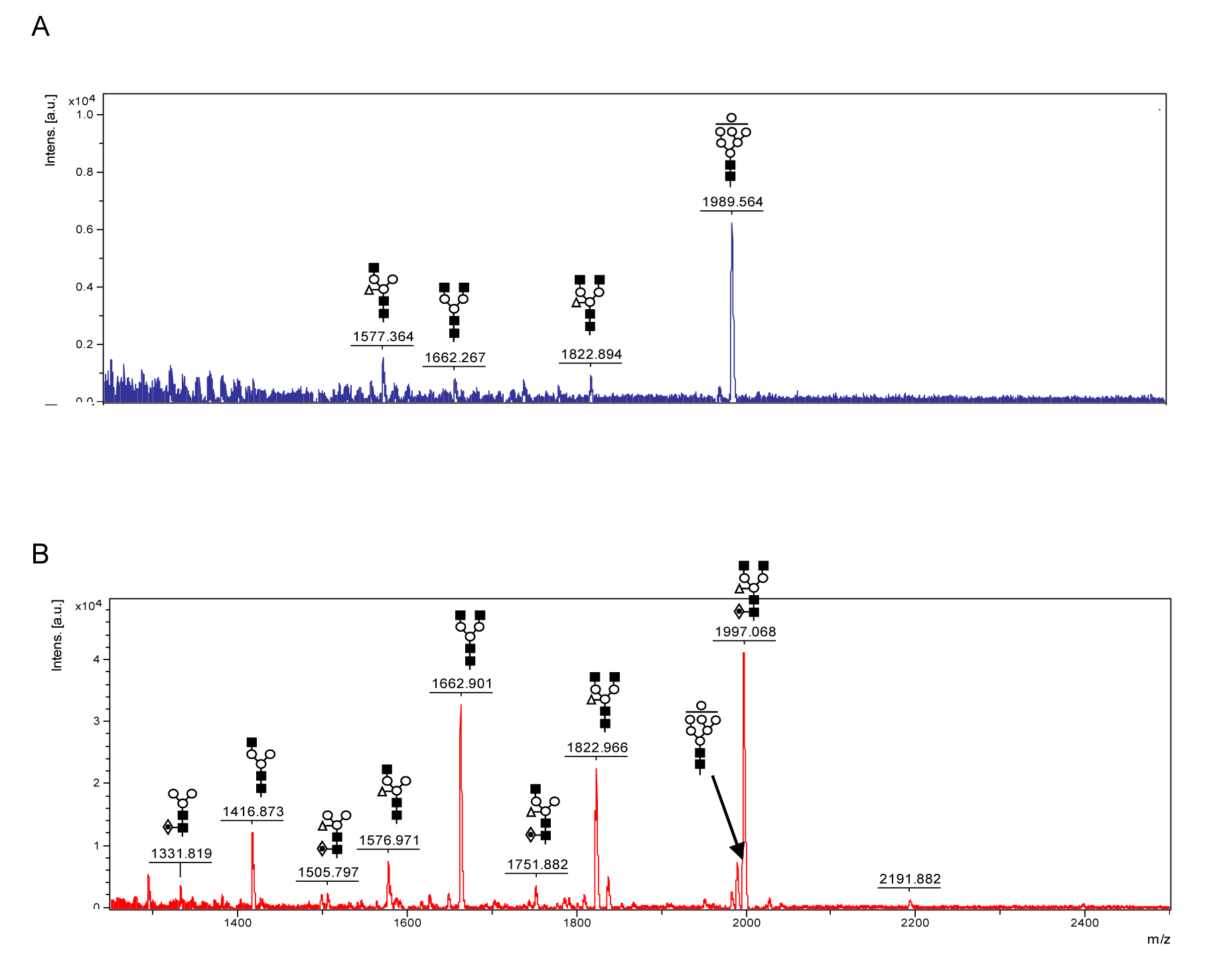

Supplement: Figure S3 — N-glycan analysis by mass spectrometry. Glycan profiles were cross-checked by mass spectrometric analysis, which provided the possible glycan structures. After permethylation for enhancing the sensitivity, the mass of glycans prepared from mAbP K (A) and mAbP (B) were analyzed. The symbols of the glycan structures are as follows: GlcNAc, black square; mannose, white circle; xylose, white triangle; fucose, diamond with a dot inside. (TIF) [file pone.0068772.s005.tif]
